# 4-Methyl-*N*-[(*S*)-1-phenyl­eth­yl]benzene­sulfonamide

**DOI:** 10.1107/S1600536810032113

**Published:** 2010-08-21

**Authors:** Zeynep Keleşoğlu, Zeynep Gültekin, Orhan Büyükgüngör

**Affiliations:** a Department of Physics, Ondokuz Mayıs University, TR-55139 Samsun, Turkey; b Department of Chemistry, Çankırı Karatekin University, TR-18100 Çankırı, Turkey

## Abstract

In the title compound, C_15_H_17_NO_2_S, the dihedral angle between the aromatic rings is 14.47 (8)°. The mol­ecule is bent at the N atom, with a C—SO2—NH—C torsion angle of 79.06 (13)°. In the crystal structure, the sulfonamide groups are hydrogen bonded *via* N—H⋯O links, forming chains of mol­ecules along the crystallographic *b* axis. π–π inter­actions [centroid–centroid distance = 3.81 (3) Å] also occur.

## Related literature

For general background to sulfonamides, see: Siddiqui *et al.* (2008 ▶) and literature cited therein; Padeiskaya & Polukhina (1974 ▶). For the anti­microbial properties of sulfonamides and their applications in medical practice, see: Mashkovskii (1987 ▶); Zhungietu & Granik, (2000 ▶). For chemical aspects of related compounds, see: Liu *et al.* (2009*a* ▶,*b* ▶); Seong *et al.* (1998 ▶). For related structures, see: Deng & Hu (2005 ▶); Zhu *et al.* (2008 ▶); Chatterjee *et al.* (1982 ▶); Ghosh *et al.* (1991 ▶); Takasuka & Nakai, (2001 ▶). For spectroscopic data for the title compound, see: Georgy *et al.* (2009 ▶).
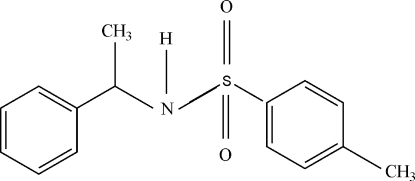


## Experimental

### 

#### Crystal data

C_15_H_17_NO_2_S
*M* *_r_* = 275.36
Monoclinic, 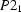

*a* = 8.1588 (4) Å
*b* = 10.1498 (4) Å
*c* = 8.9242 (5) Å
β = 105.545 (4)°
*V* = 711.98 (6) Å^3^
*Z* = 2
Mo *K*α radiation
μ = 0.23 mm^−1^
*T* = 296 K
0.76 × 0.55 × 0.38 mm

#### Data collection

Stoe IPDS 2 diffractometer
Absorption correction: integration (*X-RED32*; Stoe & Cie, 2002 ▶) *T*_min_ = 0.881, *T*_max_ = 0.933
10843 measured reflections
2950 independent reflections
2864 reflections with *I* > 2σ(*I*)
*R*_int_ = 0.027

#### Refinement

*R*[*F* ^2^ > 2σ(*F* ^2^)] = 0.029
*wR*(*F* ^2^) = 0.076
*S* = 1.05
2950 reflections
176 parameters
2 restraints
H atoms treated by a mixture of independent and constrained refinement
Δρ_max_ = 0.17 e Å^−3^
Δρ_min_ = −0.23 e Å^−3^
Absolute structure: Flack (1983 ▶), 1388 Friedel pairs
Flack parameter: −0.02 (5)

### 

Data collection: *X-AREA* (Stoe & Cie, 2002 ▶); cell refinement: *X-AREA*; data reduction: *X-RED32* (Stoe & Cie, 2002 ▶); program(s) used to solve structure: *SHELXS97* (Sheldrick, 2008 ▶); program(s) used to refine structure: *SHELXL97* (Sheldrick, 2008 ▶); molecular graphics: *ORTEP-3 for Windows* (Farrugia, 1997 ▶); software used to prepare material for publication: *WinGX* (Farrugia, 1999 ▶).

## Supplementary Material

Crystal structure: contains datablocks I, global. DOI: 10.1107/S1600536810032113/si2286sup1.cif

Structure factors: contains datablocks I. DOI: 10.1107/S1600536810032113/si2286Isup2.hkl

Additional supplementary materials:  crystallographic information; 3D view; checkCIF report

## Figures and Tables

**Table 1:** Hydrogen-bond geometry (Å, °)

*D*—H⋯*A*	*D*—H	H⋯*A*	*D*⋯*A*	*D*—H⋯*A*
N1—H16⋯O1^i^	0.84 (2)	2.11 (2)	2.9519 (17)	178 (1)

Symmetry code: (i) 
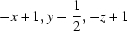
.

**Fig. 1.**
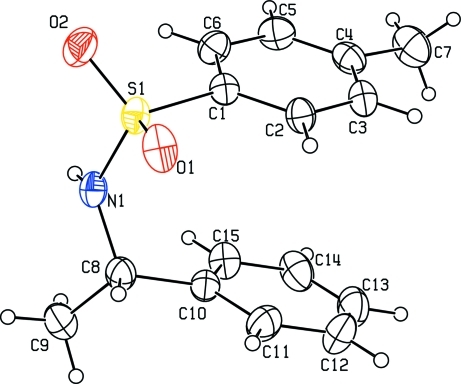
An ORTEP view of (I), with the atom-numbering scheme and 30% probability displacement ellipsoids.

**Fig. 2.**
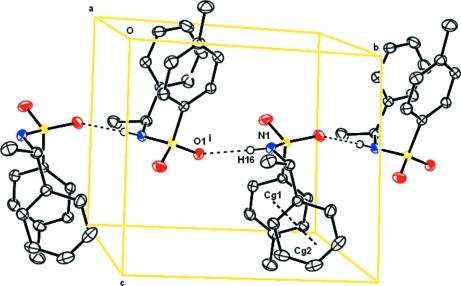
A packing diagram for (I), showing the N—H···O hydrogen bonds and π–π interactions. H atoms not involved in hydrogen bonding (dashed lines) have been omitted for clarity. [Symmetry code; (i): 1 - x, -1/2 + y, 1 - z]. (Cg1 and Cg2 are the centroids of the C1—C6 and C10—C15 rings, respectively).

## supplementary crystallographic information

### Comment

Benzenesulfonamide derivatives are well known in the biological sciences for their antibacterial, anticancer and anti- HIV activities. In the field of catalysis, their chloro derivatives are particularly important for carrying out a large number of oxidation reactions wherein the reaction kinetics are very important (Siddiqui *et al.*, 2008 and literature cited therein).

Sulfonamides possess a number of antimicrobial properties and are applied in medical practice for treating infections caused by pathogens (Mashkovskii, 1987; Zhungietu & Granik, 2000). However, considering the ubiquitous spreading of resistant forms of bacteria, the problem of preparing novel sulfonamides with advanced biomedical characteristics and the investigation of their structures and properties remain highly topical.

The benzylic and allylic amines are useful alkylating agents for the formation of carbon-carbon and carbon-heteroatom bonds under lewis acid conditions and benzylic, allylic halides and the corresponding sulfonates are frequently employed as the alkylating agents.(Liu *et al.*, 2009*a*). There are few reports for the preparation of diarylated derivatives and sulfinic acid (Liu *et al.*, 2009*b*; Seong *et al.*, 1998).

The benzene rings A(C1—C6) and B(C10—C15) are both nearly planar with the maximum r.m.s. deviation from the mean plane as 0.0067 (12)Å for C5 (Fig. 1). The *para*-substituent on the conformation of the benzene ring is nearly planar to the that of the other benzene ring, making a dihedral angle of 14.47 (8)°.

In the crystal, the molecules are linked in opposite directions with each other *via* N1—H16···O1 intermolecular interactions and π–π stacking interactions between the benzene rings (centroid to centroid distance = 3.81 (3)Å are also effective in crystal packing. (Fig. 2, Table 1).

The molecule is bent at the N atom with a C—SO2—NH—C torsion angle of 79.06 (13)° and agree with the corresponding angle -77.2 (2)° in *N*-[4-(Dimethylamino)benzylidene]-4-methylbenzene-sulfonamide (Deng & Hu, 2005). The atoms around the sulfonamide S atom is arranged in a slightly distorted tetrahedral configuration. The largest deviation is in the angle O2—S1—O1 [119.63 (8)°]. In *N*,4-Dimethyl-*N*-(4-nitrobenzyl)benzene- sulfonamide, the similar angle is 119.53 (15)° (Zhu *et al.*, 2008) and the same conforms to the non-tetrahedral nature commonly observed in sulfonamides (Chatterjee *et al.*, 1982; Ghosh *et al.*, 1991; Takasuka & Nakai, 2001).

### Experimental

(S) 1-Phenyl ethyl amine (1 g, 8.2 mmol) was dissolved in toluene (20 ml) under nitrogene at room temperature. TsCl (1.87 g, 9.8 mmol) was added to this solution. After 5 minutes the white precipate was formed. After stirring 5 minutes diisopropyl ethyl amine (1.43 ml, 8.2 mmol) was added to the solution. The reaction mixture was stirred at room temperature for an additional 3.5 h. After 3.5 h no amine was detected by TLC. The solution washed two times with water and organic layer dried over MgSO_4_. Solvent evaporated. The crude mixture purified by column chromotography PE/EtOAc (1:1), gave the title compound as a white crystalline solid (1.8 g, 79%). *M*.p. 87–89°C. Spectroskopic data identical with Lit. (Georgy *et al.*, 2009).

### Refinement

The H atom of the NH group was located in a difference map and refined freely. All other H atoms were positioned with idealized geometry using a riding model, with C–H = 0.93 Å (aromatic), 0.98 Å (methine) and 0.96 Å (methyl). All H atoms were refined with *U*_iso_=1.2*U*_eq_ (parent atom).

### Crystal data

**Table:**

C_15_H_17_NO_2_S	*F*(000) = 292
*M**_r_* = 275.36	*D*_x_ = 1.284 Mg m^−^^3^
Monoclinic, *P*2_1_	Mo *K*α radiation, λ = 0.71073 Å
Hall symbol: P 2yb	Cell parameters from 10843 reflections
*a* = 8.1588 (4) Å	θ = 2.4–26.9°
*b* = 10.1498 (4) Å	µ = 0.23 mm^−^^1^
*c* = 8.9242 (5) Å	*T* = 296 K
β = 105.545 (4)°	Plane graphite, colorless
*V* = 711.98 (6) Å^3^	0.76 × 0.55 × 0.38 mm
*Z* = 2	

### Data collection

**Table:**

Stoe IPDS 2 diffractometer	2950 independent reflections
Radiation source: fine-focus sealed tube	2864 reflections with *I* > 2σ(*I*)
graphite	*R*_int_ = 0.027
rotation method scans	θ_max_ = 26.5°, θ_min_ = 2.4°
Absorption correction: integration (*X-RED32*; Stoe & Cie, 2002)	*h* = −10→10
*T*_min_ = 0.881, *T*_max_ = 0.933	*k* = −12→12
10843 measured reflections	*l* = −11→11

### Refinement

**Table:**

Refinement on *F*^2^	Secondary atom site location: difference Fourier map
Least-squares matrix: full	Hydrogen site location: inferred from neighbouring sites
*R*[*F*^2^ > 2σ(*F*^2^)] = 0.029	H atoms treated by a mixture of independent and constrained refinement
*wR*(*F*^2^) = 0.076	*w* = 1/[σ^2^(*F*_o_^2^) + (0.0563*P*)^2^ + 0.0201*P*] where *P* = (*F*_o_^2^ + 2*F*_c_^2^)/3
*S* = 1.05	(Δ/σ)_max_ < 0.001
2950 reflections	Δρ_max_ = 0.17 e Å^−^^3^
176 parameters	Δρ_min_ = −0.23 e Å^−^^3^
2 restraints	Absolute structure: Flack (1983), 1388 Friedel pairs
Primary atom site location: structure-invariant direct methods	Flack parameter: −0.02 (5)

### Special details

**Table:**

Geometry. All e.s.d.'s (except the e.s.d. in the dihedral angle between two l.s. planes) are estimated using the full covariance matrix. The cell e.s.d.'s are taken into account individually in the estimation of e.s.d.'s in distances, angles and torsion angles; correlations between e.s.d.'s in cell parameters are only used when they are defined by crystal symmetry. An approximate (isotropic) treatment of cell e.s.d.'s is used for estimating e.s.d.'s involving l.s. planes.
Refinement. Refinement of *F*^2^ against ALL reflections. The weighted *R*-factor *wR* and goodness of fit *S* are based on *F*^2^, conventional *R*-factors *R* are based on *F*, with *F* set to zero for negative *F*^2^. The threshold expression of *F*^2^ > σ(*F*^2^) is used only for calculating *R*-factors(gt) *etc*. and is not relevant to the choice of reflections for refinement. *R*-factors based on *F*^2^ are statistically about twice as large as those based on *F*, and *R*- factors based on ALL data will be even larger.

### Fractional atomic coordinates and isotropic or equivalent isotropic displacement parameters (Å^2^)

**Table:**

	*x*	*y*	*z*	*U*_iso_*/*U*_eq_	
C1	0.69009 (17)	0.77118 (14)	0.68328 (15)	0.0428 (3)	
C2	0.67635 (19)	0.88063 (14)	0.77051 (17)	0.0476 (3)	
H2	0.6034	0.9491	0.7269	0.057*	
C3	0.7724 (2)	0.88734 (17)	0.92370 (18)	0.0535 (3)	
H3	0.7633	0.9609	0.9831	0.064*	
C4	0.8815 (2)	0.78666 (18)	0.98963 (19)	0.0553 (4)	
C5	0.8956 (2)	0.6792 (2)	0.9000 (2)	0.0609 (4)	
H5	0.9708	0.6119	0.9432	0.073*	
C6	0.8002 (2)	0.66945 (17)	0.7473 (2)	0.0546 (3)	
H6	0.8096	0.5958	0.6882	0.066*	
C7	0.9778 (3)	0.7926 (3)	1.1598 (2)	0.0814 (6)	
H7A	1.0469	0.8706	1.1789	0.098*	
H7B	1.0491	0.7163	1.1864	0.098*	
H7C	0.8984	0.7948	1.2221	0.098*	
C8	0.25591 (18)	0.69454 (16)	0.54631 (17)	0.0492 (3)	
H8	0.2031	0.7677	0.4788	0.059*	
C9	0.1317 (2)	0.5803 (2)	0.5145 (3)	0.0721 (5)	
H9A	0.1025	0.5591	0.4057	0.087*	
H9B	0.0308	0.6043	0.5437	0.087*	
H9C	0.1833	0.5050	0.5739	0.087*	
C10	0.29391 (16)	0.74216 (18)	0.71331 (15)	0.0466 (3)	
C11	0.2449 (2)	0.86701 (19)	0.7442 (2)	0.0638 (4)	
H11	0.1885	0.9212	0.6627	0.077*	
C12	0.2791 (3)	0.9121 (2)	0.8955 (3)	0.0831 (6)	
H12	0.2453	0.9964	0.9151	0.100*	
C13	0.3624 (3)	0.8335 (3)	1.0168 (2)	0.0803 (6)	
H13	0.3844	0.8639	1.1186	0.096*	
C14	0.4129 (3)	0.7105 (2)	0.9874 (2)	0.0714 (6)	
H14	0.4712	0.6575	1.0695	0.086*	
C15	0.3784 (2)	0.66387 (18)	0.83688 (18)	0.0568 (4)	
H15	0.4121	0.5792	0.8184	0.068*	
N1	0.40634 (17)	0.65258 (12)	0.49794 (14)	0.0465 (3)	
O1	0.48302 (17)	0.87632 (11)	0.44036 (12)	0.0612 (3)	
O2	0.65796 (18)	0.68619 (15)	0.40456 (14)	0.0684 (3)	
S1	0.55950 (4)	0.75139 (4)	0.49215 (3)	0.04629 (10)	
H16	0.4401 (19)	0.5744 (16)	0.5177 (19)	0.044 (4)*	

### Atomic displacement parameters (Å^2^)

**Table:**

	*U*^11^	*U*^22^	*U*^33^	*U*^12^	*U*^13^	*U*^23^
C1	0.0486 (6)	0.0399 (7)	0.0399 (6)	−0.0036 (5)	0.0121 (5)	0.0028 (5)
C2	0.0549 (7)	0.0398 (7)	0.0448 (7)	−0.0017 (6)	0.0075 (6)	−0.0004 (6)
C3	0.0611 (8)	0.0499 (8)	0.0464 (7)	−0.0085 (7)	0.0093 (6)	−0.0067 (6)
C4	0.0486 (7)	0.0667 (11)	0.0466 (7)	−0.0086 (6)	0.0058 (6)	0.0061 (6)
C5	0.0541 (8)	0.0640 (10)	0.0610 (9)	0.0129 (7)	0.0093 (7)	0.0120 (8)
C6	0.0596 (8)	0.0492 (8)	0.0561 (8)	0.0090 (7)	0.0172 (7)	0.0004 (7)
C7	0.0795 (12)	0.1006 (17)	0.0517 (9)	−0.0085 (11)	−0.0040 (8)	0.0086 (9)
C8	0.0502 (7)	0.0509 (7)	0.0418 (7)	0.0045 (6)	0.0044 (5)	−0.0014 (6)
C9	0.0593 (9)	0.0869 (14)	0.0674 (11)	−0.0175 (9)	0.0124 (8)	−0.0182 (10)
C10	0.0478 (6)	0.0465 (7)	0.0449 (6)	−0.0002 (6)	0.0113 (5)	−0.0038 (7)
C11	0.0646 (9)	0.0576 (10)	0.0690 (10)	0.0131 (8)	0.0177 (8)	−0.0074 (8)
C12	0.0906 (14)	0.0773 (14)	0.0898 (15)	0.0018 (11)	0.0388 (12)	−0.0340 (12)
C13	0.0881 (14)	0.1013 (17)	0.0577 (10)	−0.0286 (12)	0.0306 (10)	−0.0272 (11)
C14	0.0815 (11)	0.0865 (15)	0.0437 (8)	−0.0241 (10)	0.0127 (8)	0.0035 (8)
C15	0.0693 (9)	0.0506 (8)	0.0489 (8)	−0.0047 (7)	0.0131 (7)	0.0035 (7)
N1	0.0589 (6)	0.0347 (6)	0.0439 (6)	−0.0013 (5)	0.0105 (5)	−0.0056 (5)
O1	0.0858 (8)	0.0432 (6)	0.0457 (5)	−0.0070 (5)	0.0022 (5)	0.0079 (5)
O2	0.0826 (8)	0.0785 (8)	0.0519 (6)	−0.0063 (7)	0.0317 (6)	−0.0120 (6)
S1	0.06240 (19)	0.04072 (16)	0.03579 (15)	−0.00478 (15)	0.01324 (12)	−0.00162 (14)

### Geometric parameters (Å, °)

**Table:**

C1—C2	1.378 (2)	C9—H9A	0.9600
C1—C6	1.387 (2)	C9—H9B	0.9600
C1—S1	1.7638 (13)	C9—H9C	0.9600
C2—C3	1.383 (2)	C10—C11	1.379 (3)
C2—H2	0.9300	C10—C15	1.384 (2)
C3—C4	1.379 (2)	C11—C12	1.381 (3)
C3—H3	0.9300	C11—H11	0.9300
C4—C5	1.375 (3)	C12—C13	1.370 (4)
C4—C7	1.512 (2)	C12—H12	0.9300
C5—C6	1.380 (2)	C13—C14	1.362 (4)
C5—H5	0.9300	C13—H13	0.9300
C6—H6	0.9300	C14—C15	1.381 (2)
C7—H7A	0.9600	C14—H14	0.9300
C7—H7B	0.9600	C15—H15	0.9300
C7—H7C	0.9600	N1—S1	1.6138 (13)
C8—N1	1.469 (2)	N1—H16	0.843 (15)
C8—C9	1.516 (2)	O1—S1	1.4339 (12)
C8—C10	1.5177 (19)	O2—S1	1.4251 (13)
C8—H8	0.9800		
			
C2—C1—C6	120.59 (13)	H9A—C9—H9B	109.5
C2—C1—S1	121.23 (11)	C8—C9—H9C	109.5
C6—C1—S1	118.06 (11)	H9A—C9—H9C	109.5
C1—C2—C3	119.18 (14)	H9B—C9—H9C	109.5
C1—C2—H2	120.4	C11—C10—C15	118.51 (15)
C3—C2—H2	120.4	C11—C10—C8	119.70 (15)
C4—C3—C2	121.00 (15)	C15—C10—C8	121.78 (16)
C4—C3—H3	119.5	C10—C11—C12	120.45 (19)
C2—C3—H3	119.5	C10—C11—H11	119.8
C5—C4—C3	118.99 (14)	C12—C11—H11	119.8
C5—C4—C7	120.90 (17)	C13—C12—C11	120.5 (2)
C3—C4—C7	120.05 (17)	C13—C12—H12	119.8
C4—C5—C6	121.19 (16)	C11—C12—H12	119.8
C4—C5—H5	119.4	C14—C13—C12	119.55 (19)
C6—C5—H5	119.4	C14—C13—H13	120.2
C5—C6—C1	119.03 (16)	C12—C13—H13	120.2
C5—C6—H6	120.5	C13—C14—C15	120.6 (2)
C1—C6—H6	120.5	C13—C14—H14	119.7
C4—C7—H7A	109.5	C15—C14—H14	119.7
C4—C7—H7B	109.5	C14—C15—C10	120.44 (18)
H7A—C7—H7B	109.5	C14—C15—H15	119.8
C4—C7—H7C	109.5	C10—C15—H15	119.8
H7A—C7—H7C	109.5	C8—N1—S1	122.98 (10)
H7B—C7—H7C	109.5	C8—N1—H16	117.6 (11)
N1—C8—C9	106.99 (14)	S1—N1—H16	112.3 (11)
N1—C8—C10	114.46 (12)	O2—S1—O1	119.63 (8)
C9—C8—C10	112.21 (15)	O2—S1—N1	106.51 (8)
N1—C8—H8	107.6	O1—S1—N1	106.62 (7)
C9—C8—H8	107.6	O2—S1—C1	107.46 (7)
C10—C8—H8	107.6	O1—S1—C1	107.99 (7)
C8—C9—H9A	109.5	N1—S1—C1	108.18 (6)
C8—C9—H9B	109.5		
			
C6—C1—C2—C3	−0.7 (2)	C11—C12—C13—C14	0.5 (3)
S1—C1—C2—C3	175.22 (11)	C12—C13—C14—C15	−1.1 (3)
C1—C2—C3—C4	0.2 (2)	C13—C14—C15—C10	0.9 (3)
C2—C3—C4—C5	0.8 (2)	C11—C10—C15—C14	−0.2 (3)
C2—C3—C4—C7	−176.55 (16)	C8—C10—C15—C14	179.21 (15)
C3—C4—C5—C6	−1.4 (3)	C9—C8—N1—S1	172.13 (12)
C7—C4—C5—C6	175.96 (18)	C10—C8—N1—S1	−62.90 (17)
C4—C5—C6—C1	0.9 (3)	C8—N1—S1—O2	−165.68 (12)
C2—C1—C6—C5	0.2 (2)	C8—N1—S1—O1	−36.88 (13)
S1—C1—C6—C5	−175.89 (13)	C8—N1—S1—C1	79.06 (13)
N1—C8—C10—C11	122.94 (16)	C2—C1—S1—O2	145.43 (12)
C9—C8—C10—C11	−114.90 (18)	C6—C1—S1—O2	−38.50 (14)
N1—C8—C10—C15	−56.5 (2)	C2—C1—S1—O1	15.11 (14)
C9—C8—C10—C15	65.69 (19)	C6—C1—S1—O1	−168.83 (12)
C15—C10—C11—C12	−0.3 (3)	C2—C1—S1—N1	−99.93 (12)
C8—C10—C11—C12	−179.74 (18)	C6—C1—S1—N1	76.13 (12)
C10—C11—C12—C13	0.1 (3)		

### Hydrogen-bond geometry (Å, °)

**Table:**

*D*—H···*A*	*D*—H	H···*A*	*D*···*A*	*D*—H···*A*
N1—H16···O1^i^	0.84 (2)	2.11 (2)	2.9519 (17)	178.(1)

Symmetry codes: (i) −*x*+1, *y*−1/2, −*z*+1.

## References

[bb1] Chatterjee, C., Dattagupta, J. K. & Saha, N. N. (1982). *Acta Cryst.* B**38**, 1845–1847.

[bb2] Deng, L.-P. & Hu, Y.-Z. (2005). *Acta Cryst.* E**61**, o2652–o2653.

[bb3] Farrugia, L. J. (1997). *J. Appl. Cryst.* **30**, 565.

[bb4] Farrugia, L. J. (1999). *J. Appl. Cryst.* **32**, 837–838.

[bb5] Flack, H. D. (1983). *Acta Cryst.* A**39**, 876–881.

[bb6] Georgy, M., Boucard, V., Debleds, O., Zotto, C. D. & Campagne, P. (2009). *Tetrahedron*, **65**, 1758–1766.

[bb7] Ghosh, M., Basak, A. K., Mazumdar, S. K. & Sheldrick, B. (1991). *Acta Cryst.* C**47**, 577–580.

[bb8] Liu, C. R., Li, M. B., Cheng, D. J., Yang, C. F. & Tian, S. K. (2009*b*). *Org. Lett.* **11**, 2543–2545. 10.1021/ol900788r 19449883

[bb9] Liu, C. R., Li, M. B., Yang, C. F. & Tian, S. K. (2009*a*). *Chem. Eur. J.* **15**, 793–797. 10.1002/chem.200801665 19035590

[bb10] Mashkovskii, M. D. (1987). *Pharmaceuticals* Minsk, Belorussia: V.2.

[bb18] Padeiskaya E. N. & Polukhina A. M. (1974). *Novel Sulfonylamide Medicines of Prolonged Effect for Treating Infectious Diseases* Moscow: Meditsina.

[bb11] Seong, M. R., Lee, H. J. & Kim, J. N. (1998). *Tetrahedron Lett.* **39**, 6219–6222.

[bb12] Sheldrick, G. M. (2008). *Acta Cryst.* A**64**, 112–122. 10.1107/S0108767307043930 18156677

[bb13] Siddiqui, W. A., Ahmad, S., Khan, I. U., Siddiqui, H. L. & Parvez, M. (2008). *Acta Cryst.* C**64**, o286–o289. 10.1107/S0108270108009530 18451489

[bb14] Stoe & Cie (2002). *X-RED* and *X-AREA* Stoe & Cie, Darmstadt, Germany.

[bb15] Takasuka, M. & Nakai, H. (2001). *Vib. Spectrosc.* **25**, 197–204.

[bb16] Zhu, H.-Y., Wu, Z. & Jiang, S. (2008). *Acta Cryst.* E**64**, o596. 10.1107/S1600536808000330 PMC2960812 21201934

[bb17] Zhungietu, G. I. & Granik, V. G. (2000). *Basic Principles of Drug Design* Chisinau: Izd-vo MoldGU.

